# Introduction to a special issue on big data and pain

**DOI:** 10.1097/PR9.0000000000001115

**Published:** 2023-12-14

**Authors:** Georgios Baskozos

**Affiliations:** Nuffield Department of Clinical Neurosciences, University of Oxford, Oxford, United Kingdom

**Keywords:** Machine learning, Big data, Prediction, Predictive modelling, Statistical learning, Pain

## Abstract

This special issue comprised 7 articles from leaders in the field that focus on “big pain data”, the large datasets and the associated methods for data analysis that are currently emerging in pain research. This collection of articles highlights the power and potential as well as points of caution that multi-disciplinary research utilising big data and their associated methods and interpretations present for pain research.

## Abstract 2

This special issue comprised 6 articles from leaders in the field that focus on “big pain data,” the large datasets and the associated methods for data analysis that are currently emerging in pain research. This collection of articles highlights the power and potential as well as points of caution that multidisciplinary research utilising big data and their associated methods and interpretations present for pain research.

## 1. Introduction

Since Breiman published the seminal article describing the 2 competing, or rather complementary, cultures in statistical learning,^4^ 21 years have passed. In this article, he discussed and summarised, an often not explicitly admitted, practice of statistical practitioners. Sometimes, the mechanism that generates the data is considered unknown or too complex to be modelled by a relevant abstraction, so practitioners used algorithms and numerical methods, sometimes with no closed form expression, like the ubiquitous iterative least squares logistic regression, to model a relationship between input variables and the outcome with a focus on prediction. In some problems, the estimation of a certain data model that associates a set of input features to the observed outcome is simply not feasible or a simplified abstraction might have a very poor goodness of fit and explanatory power. In these cases, a data-driven algorithmic modelling approach, for example, machine learning, might be more suitable in predicting the observed outcome. These data-driven methods require lots of data to run optimisation processes to achieve good estimates for their internal weights and also require lots of data, independently collected and diverse enough to represent the population of interest, to provide robust benchmarking and validation.

We should note that in the context of this discussion, making the scope of the method, ie, prediction for machine learning, and the assumptions behind the analysis, explicit is not something outside the machine learning culture but it is rather intrinsic in it. It is also important to stress the importance of data quality and availability. Also, as by definition, these models learn associations found in the data that has been shown to them (training data) and generalise them, and given the lack of the constraints that a data model provides, data quality is even more important and models are likely to learn and generalise biases present in the data. For example, social inequalities, differential access to the health care system, and elevated risks associated with factors that cannot and should not be interpreted as causal. A careful interpretation and understanding of what data and models show is crucial for the progress of pain research in the big data era. This also brings to the forefront of the discussion ethical issues regarding data collection and the interpretation of associations. In other words, do we identify causal relationships or risk factors and commorbidities? Are they modifiable factors? direct effects or mediators? Do we see true associations or just models learned nuances in the data that are randomly correlated with the outcome? Transparent and rigorous reporting can keep interpretations within the research scope, unlock the potential for clinical applications, and present the assumptions and limitations in a fair way.

In the pain field, the proliferation of large collaborative projects that generate high-quality longitudinal datasets and the high dimensionality data collected through wearable devices and deeply phenotyped cohorts showcased the versatility and effectiveness of machine learning and accelerated the application and development of new algorithms. Large biobanks and community-based cohorts that are longitudinally followed up provide an excellent opportunity to train predictive models and discover multiple, collectively important but sometimes individually weak associations with pain phenotypes that can lead to patient stratification and early diagnosis and treatment. Particularly, in the pain field, where phenotypes are complex, large datasets and methods that effectively facilitate their analysis highlight the need for phenotypic harmonisation and standardisation. In this context, machine learning methods can be invaluable, providing dimensionality reduction and feature selection in smaller, deeply phenotyped datasets with hundreds of data points per individual. In the coming years, we expect to see a significant increase in machine learning applications in the pain research, harvesting the increased data availability, but we also expect to see a leap in the clinical importance of these applications. These developments are expected due to advancements in methods, more rigorous modelling and careful applications, but also due to better data quality, better phenotypic definitions and harmonisation.

In this special issue, we aimed to capture and present the current state of big data analysis and machine learning applications in the pain field, highlight potentials and drawbacks, provide guidance on application and methodologies and discussing the useful tensions, ethical issues, and challenges.

This special issue starts with a comprehensive overview of the current state in machine learning (ML) applications in pain research. As big data are becoming increasingly available, predictive modelling approaches and ML in particular, are increasingly being applied to pain-related data. Lotch et al.^10^ discuss the current advances and present an overview of methods and examples of applications while highlighting appropriateness, advantages, and limitations. A data scientometric analysis presented in the article provides insights on the increased usage of ML, the most common approaches and algorithms of choice, the sample sizes being used, the input feature space, and primary endpoints of pain-related ML applications.

Despite the high prevalence and the significant burden of chronic pain in quality of life, the condition is often underdiagnosed and current treatment approaches might be inadequate.^14^ Large longitudinal cohorts and epidemiological studies have identified risk factors associated with the development of chronic pain,^1–3,9,13^ some of which are modifiable lifestyle parameters. Fundoiano-Hershcovitz et al.^7^ present a framework for a digital therapeutics approach that aims to change the patient's behaviour in order to assist and promote better pain management. Machine learning is intertwined with personalised medical interventions, based on big data, such as the data obtained by wearable devices. The approach presented here is utilising a postural biofeedback wearable device and delivers personalised training recommendations in order to assist with low back pain management. Machine learning is being used to predict long-term treatment efficacy based on individual patient characteristics. They show that pain levels were effectively reduced within 3 weeks from starting the digital therapeutics intervention, and age and gender interactively modulated pain levels and posture quality.

In this special issue, we wanted to highlight that analysis frameworks are intertwined with the process that generates data and its respective quality. The availability of publicly accessible databases, self-reported community-based cohorts, and data from wearables have created an increased interest on the methods and qualities of this “real-world evidence” (RWE) data, which has been collected outside the standardised clinical environment. In this systematic review, Vollert et al.^15^ discuss the methodological approaches required to design and analyse RWE studies in pain research. They address the usefulness, particularly when randomised control trials are very challenging to conduct, potentials, and challenges of using RWE in assessing the effectiveness of pain treatments. Based on this qualitative review, they propose good practices by advocating the early consultation of statisticians and epidemiologists, analytical transparency, the standardisation of terminology, the wider usage of rigorous causal inference methods, and highlighting the fact that this is an emerging field where there is still room for methodological improvements and novelty.

The application of ML and, in general, predictive modelling approaches in pain research have proliferated mainly due to the existence of large longitudinally followed pain-related cohorts, ie, “big pain data.” Hebert et al.^8^ present an overview, showcasing future potential, by discussing past achievements and challenges involved in large-scale pain-related cohorts aiming to untangle the complex interplay of genetic and environmental risk factors that lead to the development of chronic pain. From a public health perspective, the overarching aim is to identify the risk early and facilitate interventions to prevent the transition of nociceptive or transient pain to severe chronic pain. The challenges arise from the heterogeneity of the disorder and the response to treatment. In addition, research approaches are hindered from the lack of phenotypic harmonisation and standardisation of data collection principles, sampling biases, lack of representativeness, and relatively small sample sizes. The authors discuss the outputs, advancements, caveats, and lessons learned from large collaborative research initiatives such as the DOLORisk,^11^ PAINSTORM, and biorepositories such as the UK biobank.^12^ They also present a way forward that includes large national-level biobanks alongside smaller deeply phenotyped cohorts that combined with the rapid advances in multiomics and neural circuit-level approaches can eventually fulfil the urgent need for the development of novel analgesics.

As it has already been pointed out, small sample sizes and heterogeneity in the pathogenesis of chronic pain call for methodological approaches that can increase power by integrating data. Fundaun et al.^6^ present a guide, accompanied by examples, for the design, planning, and conducting of meta-analysis. They present and discuss the advantages and disadvantages of the most commonly used meta-analysis models and evaluate the clinical implications of meta-analysis in pain research. This article addresses the need to obtain larger sample sizes and, as a consequence, higher precision estimates using the expanding pool of available pain related data, but it can also be read as a companion to the researcher for the interpretation of meta-analysis results and the selection of the most appropriate method.

We close this special issue with a perspective article from Crombez et al.,^5^ opening a discussion on the advancements needed in the research of the effects of psychological factors on pain outcomes. The authors start by acknowledging the potentials of the big data era and challenging and highlighting the methods and assumptions, sometime implicit, in researching psychological factors of pain. But they do not limit their scope on psychological factors only. They rather bring forward the much needed discussion on data quality, construct validity, and causal assumptions, particularly in the era of “off-the-self” black box algorithms. They call for a more rigorous approach that makes assumptions explicit and reporting more transparent. This call back to the “drawing board” can be valuable for both the clinician and the data analyst in unlocking the potential of big data and machine learning with clinically relevant outcomes in pain research. Regarding the ongoing discussion investigating the cause–effect relationship between pain and psychosocial factors, they stress the importance of respecting the 3 basic tenets of causality, namely, (1) cause and effect differ from each other, (2) the cause precedes the effect within reasonable time, and (3) alternative explanations are ruled out.

Closing this special issue, we wanted to show that there is indeed an important crosstalk between the 2 cultures of statistical learning,^4^ the potential of applying powerful and complex machine learning algorithms to predict outcomes, and the need for a rigorous causal modelling to proceed with causal inference or the building of large national-level databases and the utilisation of smaller deeply phenotyped cohorts. These are only a small part of the constructive discussions and productive tensions that are the moving power of scientific advancement.

## Disclosures

The author has no conflict of interest to declare.
